# Can immunocrit be used as a monitoring tool for swine vaccination and infection studies?

**DOI:** 10.1186/s40813-024-00380-y

**Published:** 2024-08-23

**Authors:** Mònica Sagrera, Marina Sibila, Núria Martínez-Boixaderas, Anna Maria Llorens, David Espigares, Josep Pastor, Laura Garza-Moreno, Joaquim Segalés

**Affiliations:** 1 IRTA, Programa de Sanitat Animal, Centre de Recerca en Sanitat Animal (CReSA), Campus de La UAB, 08193 Bellaterra (Cerdanyola del Vallès), Spain; 2 Unitat Mixta d’Investigació IRTA-UAB en Sanitat Animal, Centre de Recerca en Sanitat Animal (CReSA), Campus de la Universitat Autònoma de Barcelona (UAB), 08193 Bellaterra, Barcelona, Spain; 3 Ceva Salud Animal, Avenida Diagonal, 08017 Barcelona, Spain; 4 WOAH Collaborating Center for Research and Control of Emerging and Re-Emerging Pig Diseases in Europe (IRTA-CReSA), 08193 Bellaterra, Barcelona, Spain; 5 Departament de Sanitat i Anatomia Animals, Facultat de Veterinària, UAB, 08193 Bellaterra, Barcelona, Spain

**Keywords:** Immunocrit, Monitoring, Vaccination, Infection, Porcine circovirus 2, Total serum protein

## Abstract

**Background:**

The immunocrit is a cost-effective and straightforward technique traditionally used to assess passive immunity transfer to newborn piglets. However, it has not been previously used for monitoring the effect of vaccination and/or infections. Therefore, this study aimed to evaluate the usefulness of the immunocrit technique as an immunological monitoring tool in a vaccination and challenge scenario, using porcine circovirus 2 (PCV-2) as pathogen model. The immunocrit ratio was monitored in PCV-2 vaccinated (V) and non-vaccinated (NV) 3-week-old piglets (study day 0, SD0) that were subsequently challenged with this virus at SD21 and followed up to SD42. Additional techniques (PCV-2 IgG ELISA, optical refractometry, and proteinogram) were performed to further characterize the results of the immunocrit analysis.

**Results:**

Immunocrit, γ-globulin concentration and PCV-2 S/P values followed similar dynamics: descending after PCV-2 vaccination but ascending after an experimental PCV-2 inoculation. However, statistically significant differences between V and NV animals were only found with the PCV-2 ELISA. In this case, V animals had significantly higher (*p* < *0.05*) S/P values (S/P ratio = 0.74) than NV (S/P ratio = 0.39) pigs only after challenge at SD42. On the other hand, serum total protein obtained by refractometer (STPr) were maintained from SD0 to SD21 and increased in both groups from SD21 to SD42. Correlations between techniques were low to moderate, being the most robust ones found between immunocrit and optical refractometry (ρ = 0.41) and immunocrit with γ-globulins (ρ = 0.39). In a subset of sera, the proteinogram technique was applied to the whole serum and the supernatant of the immunocrit, with the objective to characterize indirectly the immunocrit fraction. The latter one included all protein types detectable through the proteinogram, with percentages varying between 64.3% (γ-globulins) and 82% (β-globulins).

**Conclusion:**

The immunocrit technique represented a fraction of the total serum proteins, with low to moderate correlation with all the complementary techniques measured in this study. Its determination at different time points did not allow monitoring the effect of vaccination and/or infection using PCV-2 as a pathogen model.

**Supplementary Information:**

The online version contains supplementary material available at 10.1186/s40813-024-00380-y.

## Background

The advancements in pig breeding genetics, resulting in highly prolific sows and larger litters with consistent weight differences at birth, pose a critical challenge for the swine production [12]. Piglets are born immunologically incompetent due to the epitheliochorial placental structure, which impedes the transfer of immune cells and antibodies to the foetus during gestation [35]. Consequently, piglets acquire passive immunity solely through colostrum ingestion within the initial 24 h after farrowing [7, 35]. It is essential to note that the piglet's gut barrier seals between 24 to 36 h after farrowing and, subsequently, immunoglobulin (Ig) absorption is no longer guaranteed [20, 26]. Colostrum contents milk and other elements of blood plasma, such as antibodies (specially IgG) [5, 29]. However, certain piglets fail to consume a proper amount of colostrum, as well as some dams fail to initiate its production or it is insufficient, which make piglets more susceptible to infections during the first weeks of life [8, 26, 31–33].

The immunocrit is an economically viable and easy-to-perform technique traditionally used to roughly measure Ig within the first days of life from piglets [30], and other species such as calves and foals [2, 24, 25, 27], reflecting the colostrum intake. This measurement allows the implementation of strategies such as cross-fostering to improve the amount and uniformity of colostrum that receives each littermate [16, 32]. Notably, immunocrit has not been evaluated beyond the first days of life in pigs, neither other species.

In pigs, the decay of maternally derived immunity (MDI), although variable among pathogens, is generally expected to occur in the first weeks of life [22, 23, 34]. The transfer of MDI during the first days of life has been assessed using different methodologies apart from immunocrit, such as optical refractometry, enzyme-linked immunosorbent assay (ELISA), and the proteinogram [9, 15, 26]. Vaccination and/or infection are processes that will influence the Ig levels, which depend also on factors such as level and duration of maternally derived antibodies (MDA), antigen load, vaccine type, and genetics, among others [1]. These variations have been widely studied for vaccination and/or infection against specific pathogens using ELISA, but less frequently with proteinogram technique due to their associated cost [10, 26].

Optical refractometry measures the serum total protein (STP), offering a very rough estimate of the total Ig concentration. In serum samples from neonatal piglets (after colostrum intake) Igs represent the major fraction (> 50%) of STP [4, 9, 17, 26]. In contrast, the proteinogram technique provides specific concentrations of STP (STPp) in five protein fractions: albumin and α-1, α-2, β, and γ-globulins [28].

The present study aimed to assess the immunocrit values of pigs at weaning and compare their evolution post-vaccination and subsequent viral challenge under experimental settings, using *Porcine circovirus 2* (PCV-2) as pathogen model. Immunocrit results were further characterized by means of proteinogram and optical refractometry.

## Materials and methods

### Experimental study design and sample analysis

**Fig. 1:**
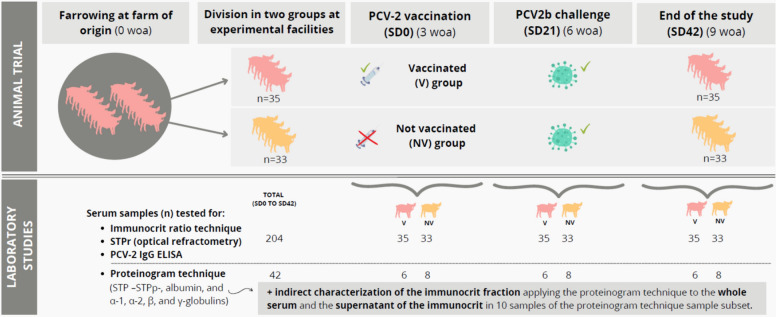
Scheme of the study design. NV: not vaccinated (received PBS intramuscularly); V: vaccinated; SD: study day; STP: serum total protein; woa: weeks of age

The experimental study design is described in Fig. 1. Seventy-two clinically healthy and PCV-2 qPCR negative piglets from non-PCV-2 vaccinated sows during gestation were selected at 2 weeks of age from their farm of origin. Usual farm practices were to vaccinate sows against *Actinobacillus pleuropneumoniae* three weeks before farrowing and a combined immunization against *Erysipelothrix rhusiopathiae and Porcine parvovirus* during lactation. The health status of the farm of origin included a positive stable status for *Porcine reproductive and respiratory syndrome virus* and seropositivity against *Mycoplasma hyopneumoniae*, *Actinobacillus pleuropneumoniae*, and *Glaesserella parasuis*.

At 3 weeks of age (study day 0 [SD0]), half of the animals were vaccinated with a PCV-2 inactivated vaccine (Circovac®, Ceva) following manufacturer instructions, and the other half were injected with phosphate buffered saline (PBS), resulting in 2 groups: vaccinated (V) (n = 36), and not vaccinated (NV) (n = 36), respectively. Three weeks after vaccination (SD21), all animals were challenged with 3 mL of inoculum 10^4^–10^5^ TCID_50_/mL of PCV-2b strain Sp-6-11-49-16. The study finished three weeks after challenge, at SD42. Serum samples were obtained at SD0, SD21 and SD42. Four animals (3 from NV group and 1 from V group) died between SD0 and SD21 due to polyserositis and meningitis associated to *Streptococcus suis*, and unrelated to the vaccination. This study was approved by the Ethics Commission of Generalitat de Catalunya (Spain) under the reference CEA-11397.

From the 68 animals that finished the study, a total of 204 serum samples from three different time-points (SD0, SD21 and SD42) were tested by immunocrit, PCV-2 IgG ELISA and optical refractometry. Additionally, a sample subset (n = 42, 20% of the total samples) was subjected to a proteinogram analysis. These 42 sera samples belonged to SD0, SD21 and SD42 of 6 randomly selected animals of the V group (n = 18) and 8 randomly selected animals of the NV group (n = 24). Finally, the supernatant after performing the immunocrit technique of 10 randomly selected samples of the subset (3 from SD0, 3 from SD21 and 4 from SD42) was additionally subjected to proteinogram analysis to compare the protein content before and after immunocrit precipitation, thereby elucidating the proportion of protein precipitated by ammonium sulphate.

### Immunocrit technique

Sixty microliters of serum of each sample (n = 204) were vortexed and mixed with 60 µL 40% (NH_4_)_2_SO_4_ (Merck, Darmstadt, Germany) following a previously published protocol [30]. Each sample was centrifuged at maximum speed (12.000 rpm) (Centromix II–BL, JP Selecta, Abrera, Spain) in a haematocrit microcapillary tube (Fisher Scientific, Waltham, MA, USA) for 5 min. The immunocrit ratio was determined by dividing the length of the precipitate in the tube with the length of the solution in the tube [30]. This calculation was performed using the Hawksley microhaematocrit reader (Hawksley, Sussex, England). Negative controls were assessed by mixing 60 µL of serum with 60 µL of distilled water and centrifuging in a haematocrit tube, and also mixing 60 µL of distilled water with 60 µL 40% (NH_4_)_2_SO_4_.

### Indirect ELISA to detect anti-PCV-2 IgG antibodies

PCV-2 antibodies were detected from serum samples, at SD0 (n = 68), SD21 (n = 68) and SD42 (n = 68), using an indirect commercial ELISA assay (Ingezim Circo IgG 11.PCV.K1® assay, INGENASA, Madrid, Spain), following manufacturer’s instructions. All serum samples from each animal were run on the same ELISA plate. The optical density (OD) was measured at 450 nm by the Sunrise™ reader (Tecan, Männendorf, Switzerland). ELISA results were expressed as mean S/P ratio (OD of sample/OD of positive control for each ELISA plate) per tested animal.

### Optical refractometry

The STP by optical refractometry (STPr) was measured in all the 204 serum samples. Briefly, 80 µL serum (previously vortexed) were added to on an optical refractometer (Euromex®, Arnhem, Netherlands) and an operator (always the same) read the STPr value from the integrated graduated scale (grams of total solid/dL of solution). Before start, the refractometer was calibrated with distilled water, and between each sample, it was cleaned with tap water.

### Proteinogram technique

The proteinogram was performed in the previously mentioned subset of 42 samples using 250 µL of serum and measured by capillary electrophoresis (MiniCap Flex Piercing, Lisses, France) at the Veterinary Biochemical Service (UAB, Bellaterra, Spain). This technique detects the STPp, which determines the amount and percentage of albumin and α-1, α-2, β, and γ-globulins. The STPs was detected using the OSR (Olympus System Reagent®, Beckman Coulter, Pasadena, CA, USA) reagent for the Biuret method and analysed by an Olympus AU480 machine (Beckman Coulter, Pasadena, CA, USA).

To determine the percentage of protein precipitated after ammonium sulphate (immunocrit technique), 300 µL of the previously mentioned sample subset (n = 10) was vortexed and mixed with 300 µL of 40% (NH_4_)_2_SO_4_ in 1.5 mL tubes (Eppendorf®, Hamburg, Germany) [30]. Samples were centrifugated at 12,700 g during 5 min (Eppendorf® Centrifuge 5425 R, Hamburg, Germany) [30]. A total of 250 µL of the supernatant were aspirated using a pipette and introduced into a new 1.5 mL tube, and the proteinogram technique was subsequently performed.

### Statistical analyses

The normality of all studied quantitative variables (immunocrit ratios, PCV-2 ELISA S/P ratios, STPr, STPp, albumin, α-1, α-2, β, and γ-globulins concentration) was checked for each group and time-point by the Shapiro Wilk’s test. Immunocrit ratio, STPr concentration and PCV-2 IgG ELISA S/P ratio differences between study days (SD0, SD21 and SD42) were analysed with the non-parametric Kruskal–Wallis test and Dunn’s multiple comparison test, and between V and NV groups with the non-parametric Mann–Whitney test. Differences on STPp, albumin, and α-1, α-2, β, and γ-globulins concentration between study days were analysed with the parametric ANOVA one way test and Tukey’s multiple comparisons test, and the t-test was used to compare between V and NV groups.

Spearman correlation coefficient (ρ) was calculated and a matrix was performed to study the correlation between techniques (immunocrit ratio, STPr, PCV-2 IgG ELISA S/P ratio, STPp, and globulins concentration). Moreover, second-order polynomial regressions were performed to explore the non-linear relationship (Draper and Smith, 1998) between immunocrit ratio and the following parameters: STPr, STPp and γ-globulins concentrations and PCV-2 IgG ELISA S/P ratio. Additionally, the second order polynomial regression was also performed between PCV-2 IgG ELISA S/P ratio and γ-globulins concentration, and between STPr and STPp concentrations. Statistical analyses and graphics were performed with Graphpad® (La Jolla, CA, USA). The significance level (*p*-value) was set at 0.05.

## Results

### Immunocrit

**Fig. 2:**
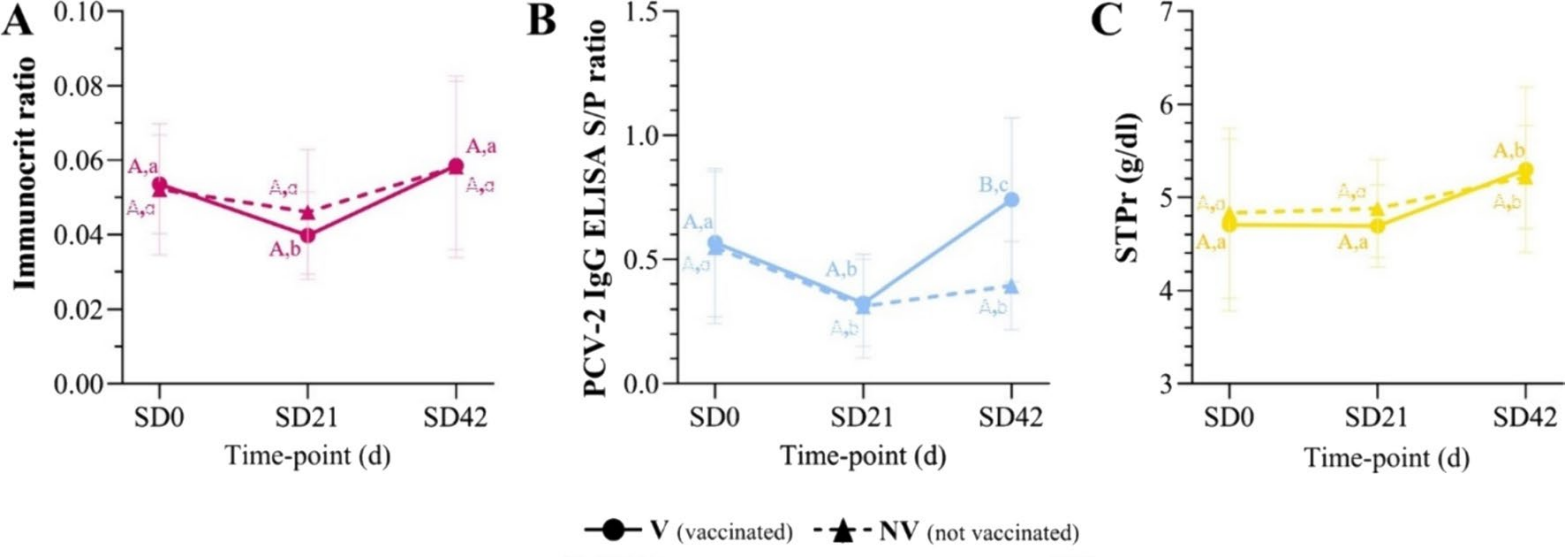
Results of the performed techniques at different time-points for V and NV animals. **A** immunocrit, **B** PCV-2 IgG ELISA S/P ratio, and **C** STPr (obtained by optical refractometry). For each technique, different uppercase letters indicate statistically significant differences between V and NV for each particular study day, and the lowercase ones indicate statistically significant differences within a group between study days (p < 0.05). V: vaccinated; NV: not vaccinated; SD: study day; STP: serum total protein

Immunocrit ratio averages for V and NV groups are shown in Fig. 2A. Both V and NV groups showed a descending dynamic after vaccination (from SD0 [vaccination] to SD21[challenge]) and an increasing one after PCV-2 infection (from SD21 to SD42 [necropsy]). Indeed, no statistically significant differences were detected when comparing groups at any sampling point. In contrast, a significantly lower immunocrit ratio (*p* < 0.05) was observed in V pigs at SD21 compared to SD0 and SD42.

### PCV-2 antibody levels measured by PCV-2 IgG ELISA

PCV-2 ELISA S/P ratios for V and NV pigs are depicted in Fig. 2B. In both groups, the S/P ratio dynamics from SD0 (vaccination) to SD21 (challenge) was descendent (*p* < 0.05). After PCV-2 challenge, S/P level from the V group increased significantly from SD21 to SD42 (*p* < 0.05), being significantly higher (*p* < 0.05) than the one from the NV group.

### Serum total protein determination by optical refractometry

The STPr average results from V and NV groups followed a similar pattern (Fig. 2C). Moreover, these values were not significantly different between SD0 and SD21 for both groups but increased significantly by SD42.

### Serum total protein, albumin and globulins concentration determined by the proteinogram technique

**Fig. 3:**
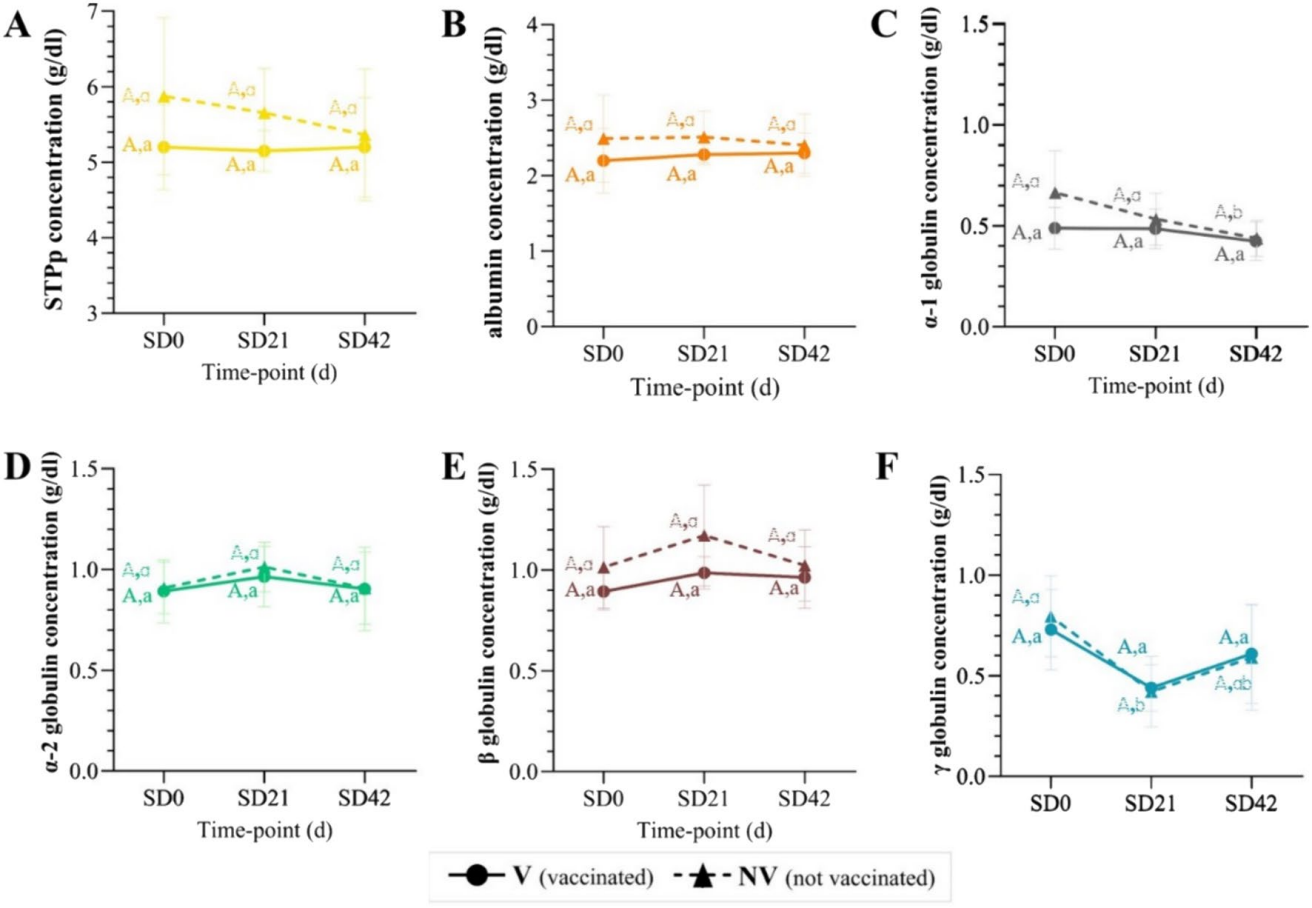
Results of different parameters obtained with the proteinogram technique at different time-points for V and NV animals. **A** STPp, **B** albumin, **C** α-1 globulin, **D** α-2 globulin, **E** β globulin, and **F** γ globulin concentration (g/dl). For each parameter, different uppercase superscript letters indicate statistically significant differences between V (bold) and NV (dotted) for each particular study day, and the lowercase ones indicate statistically significant differences within a group between study days (*p* < 0.05). V: vaccinated; NV: not vaccinated; SD: study day; STPp: serum total protein obtained by proteinogram technique

No differences were observed between V and NV groups, neither between study days for STPp (Fig. 3A) and albumin (Fig. 3B). However, α-1 globulins decreased significantly from SD21 to SD42 (p < 0.05), but only in the NV group (Fig. 3C). α-2 (Fig. 3D) and β globulins (Fig. 3E), did not show any significant change along the study period, but γ globulins showed a descending dynamic in both groups (being only significant for the NV group) from SD0 to SD21 and an ascending one from SD21 to SD42 (Fig. 3F).

### Correlation and regression analysis between parameters

**Fig. 4:**
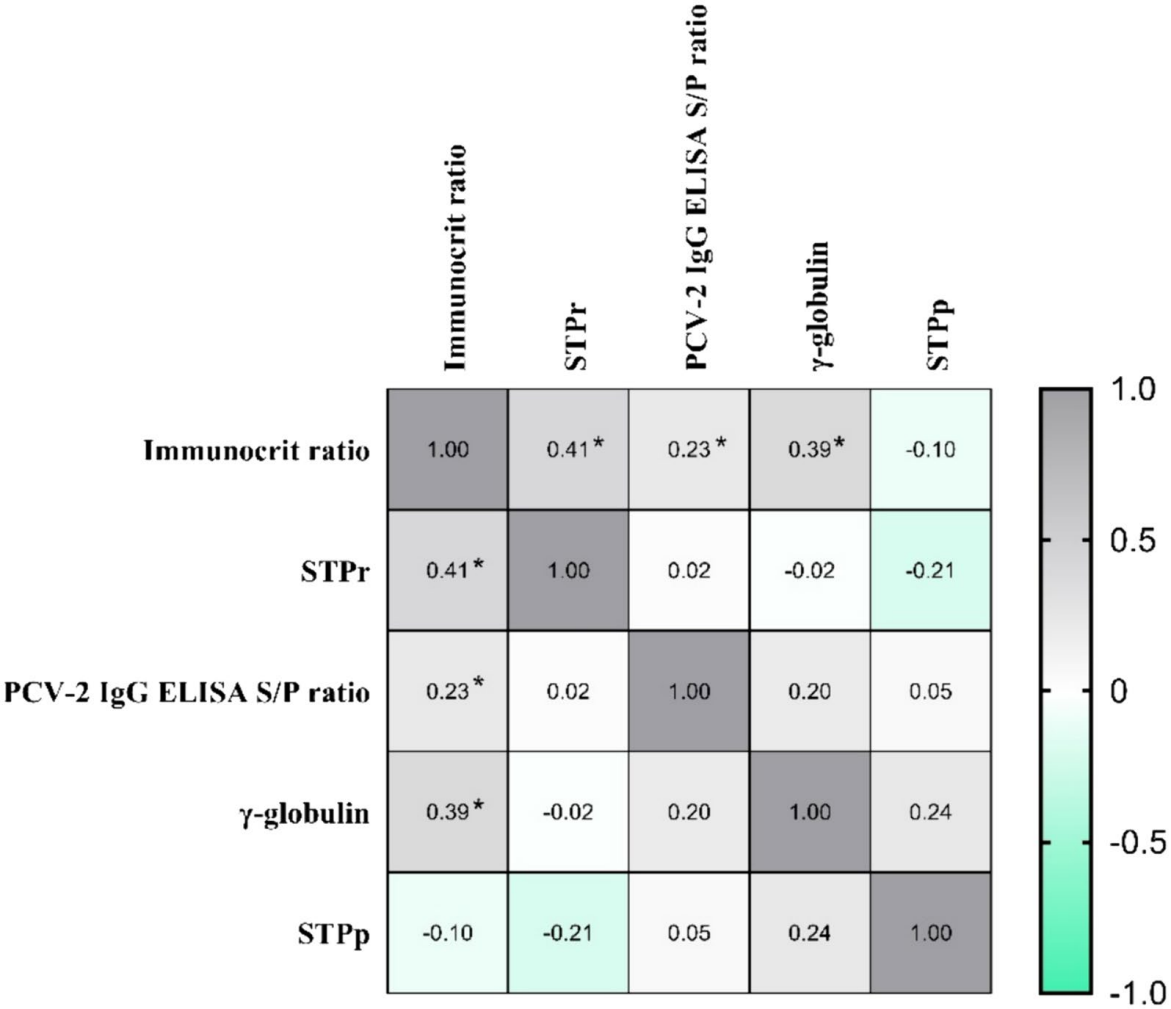
Spearman’s correlation matrix between parameters: immunocrit ratio, STPr, PCV-2 IgG ELISA S/P ratio, γ-globulin concentration and STPp. STPp: serum total protein obtained by the proteinogram technique; STPr: serum total protein obtained by optical refractometry. The asterisk symbol (*) indicates that the correlation among the analyzed parameters is statistically significant (*p* < 0.05)

Correlations and regression between studied parameters are shown in Fig. 4 and *Supplementary Fig. 1*, respectively. The highest correlation values were obtained between immunocrit ratio and STPr (ρ = 0.41, *p* < *0.05*) (Figs. 4* and Supplementary Fig. 1A*), followed by immunocrit versus γ-globulin concentration (ρ = 0.39, *p* < *0.05*) (Figs. 4 and *Supplementary Fig. 1B*). The correlation of immunocrit ratio vs. PCV-2 IgG ELISA S/P ratio was low (ρ = 0.23, *p* < *0.05*), as well as the one obtained between STPp vs. STPr concentrations (ρ = -0.21, *p* > *0.05*), PCV-2 IgG ELISA S/P ratio vs. γ-globulin concentration (ρ = 0.20, *p* > *0.05*) and immunocrit ratio vs. STPp (ρ = − 0.10, *p* > *0.05*) (Fig. 4). The R-squared values for the polynomial regressions were low (0.02–0.33) for all of them (*Supplementary Figs. 1C, 1D, 1E and 1F*).

### ***Composition of the supernatant and precipitated fraction after (NH***_***4***_***)***_***2***_***SO***_***4***_*** treatment of serum for the immunocrit analysis***

**Fig. 5:**
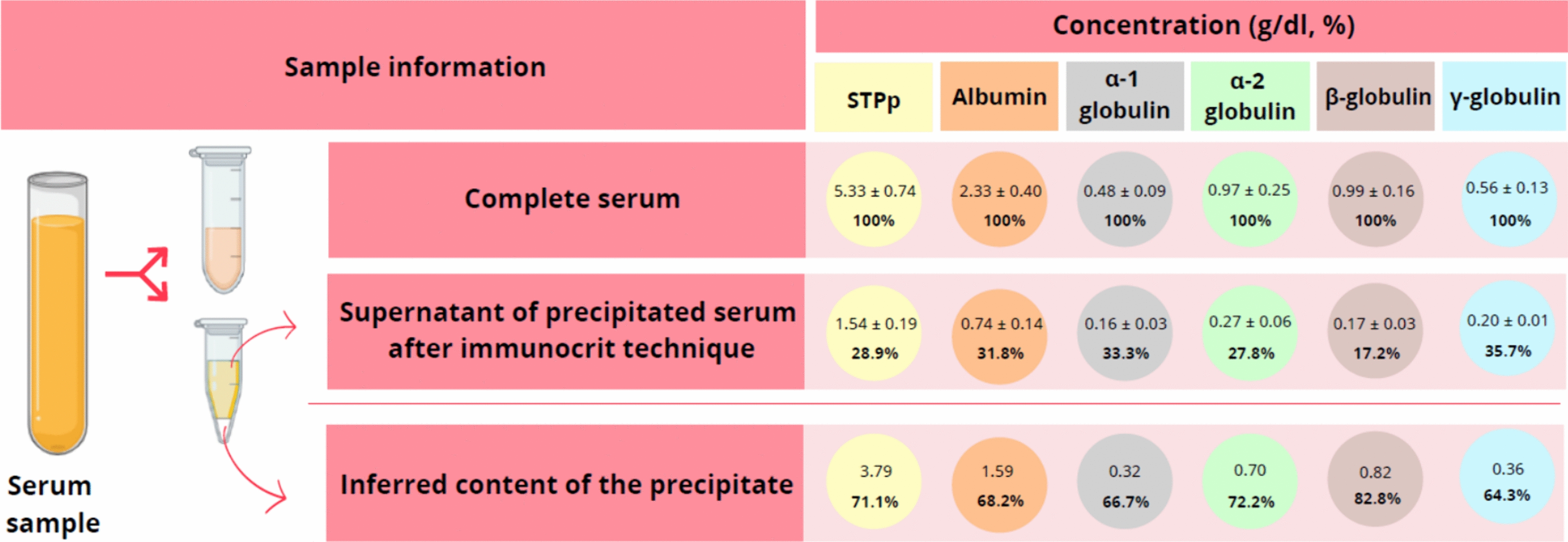
Comparison of protein concentrations (measured by the proteinogram technique) of complete serum (prior precipitation) and the supernatant after immunocrit technique (following precipitation with 40% (NH_4_)_2_SO_4_). Protein concentrations of the precipitate were inferred by substracting the protein concentration of the supernatant (17.2–35.7%) from the protein concentration of the complete serum (100%). Concentration values are expressed g/dl

The supernatant (non-precipitated fraction) of the serum after immunocrit analyses contained 28.9% of the STPp (1.54 ± 0.19 g/dL), which indicate that only the 71.1% of the STP was precipitated when the immunocrit analyses was performed (Fig. 5). The immunocrit supernatants contained different proportion of STP components, being 35.7% of γ-globulins. This value indicated that the immunocrit precipitate contained only 64.3% of the total γ-globulins present in the serum (Fig. 5). Therefore, the precipitate of the immunocrit is composed by approximately two thirds of each of the protein fractions found in a serum proteinogram.

## Discussion

The present study assessed for the first time the use of the immunocrit technique as a monitoring tool after vaccination and infection in pigs, employing PCV-2 as a pathogen model. Considering that immunocrit is used as a proxy for colostrum intake (and indirectly as a rough estimation of the MDA amount) [30], its further use at older ages was evaluated, mainly in the context of monitoring the outcome of an experimental setting including vaccination and infection. To enhance the comprehension of the obtained immunocrit results, complementary techniques to detect specific Igs (PCV-2 IgG ELISA) and STP (STPr and STPp) as well as their fractions (proteinogram), were performed.

The descending dynamics of immunocrit and PCV-2 antibodies from SD0 to SD21 in V and NV groups may be attributed to the normal decay of MDA. Specifically, such decline has been documented to occur between 4 and 12 weeks of age for PCV-2 antibodies [13, 21, 22]. This decrease of PCV-2 specific IgGs at SD21 (following PCV-2 vaccination for the V group and PBS intramuscular injection for the NV group) was comparable in both groups, which aligns with findings from previous studies where immunoglobulin levels (using similar vaccine formulation and vaccination ages) decreased during the few weeks after immunization [14, 19, 21]. However, 21 days after PCV-2b experimental challenge (SD42), only the V group revealed PCV-2 IgG seroconversion by ELISA, while both V and NV groups showed an increasing trend of immunocrit values. This fact may explain why despite similar dynamics between PCV-2 IgG ELISA and immunocrit ratio results before the challenge, the overall correlation between both techniques was low (ρ = 0.23). Such low correlation would suggest a poor biological association between both variables. Multiple reasons may account for this result, being the main one that the mentioned ELISA detects PCV-2 specific IgGs, while immunocrit detects generic Igs based on the literature [26, 30]. Indeed, for a given immunocrit ratio value, multiple corresponding PCV-2 IgG ELISA S/P ratio values were observed. In consequence, obtained results point out that immunocrit is a poor proxy for antibody monitoring as well as the other way around.

A descending γ-globulin dynamics between 2 and 6 weeks of age has been reported in another study using the proteinogram technique [29]. In the present study, the evolution of average γ-globulin concentrations and immunocrit ratios was similar (although with a low correlation of individual values) across all time-points in both V and NV groups. As previously mentioned, immunocrit roughly measures generic Igs [30]. However, when the immunocrit concentrate was studied by means of the proteinogram technique, it was found that measures a proportion of STP containing approximately 64% of total γ-globulins and a variable proportion between 67 and 83% of other serum proteins. Therefore, care must be taken when considering the immunocrit as a measure of Igs, since significant variations of other serum proteins may account for variability in the immunocrit value. Moreover, given that γ-globulins encompass other Igs such as IgA, IgE, IgG, and IgM [3, 6] and immunocrit includes a proportion of all Igs (not only IgG) as well as other serum proteins, it should be expectable that the effect of a single vaccination (inactivated vaccine in the present case) is probably negligible at modifying significantly the immunocrit and γ-globulin values.

Although low, the immunocrit ratio had the highest overall correlation with the results of the STPr when compared with all used techniques. Such correlation should not be surprising considering that immunocrit roughly measures 60–80% of the STP, being therefore, a plausible biological association. Amazingly, when a more precise technique such as the proteinogram [18] is used, correlation with immunocrit was even negative. This result was unexpected on beforehand, but not so surprising when confirming that the correlation between STPr and STPp had also a negative correlation. It must be noted that refractometer scales are typically calibrated for serum of human and/or companion animals (i.e. dog, cat), assuming that non-protein solutes like electrolytes are at similar and relatively low concentrations across species [18]. Additionally, variations in other substances like lipids, which can alter solution density, could contribute to the inaccuracy of STPr assessments [18].

## Conclusion

Obtained results indicated that the immunocrit technique did not allow monitoring groups of pigs under vaccination and/or infection scenarios, at least using PCV-2 as a model pathogen. Although variation of this parameter in postweaning pigs was subtle, it decreased slightly by 3 weeks after vaccination, and increased 3 weeks post-challenge. Further analysis of the immunocrit showed that this ratio reflects approximately two thirds of the total γ-globulins in serum.

### Supplementary Information

**Additional file 1**.

## Data Availability

All data generated or analysed during this study are included in this published article [and its supplementary information files].

## Abbreviations

ELISA: Enzyme-linked immunosorbent assay
Ig: Immunoglobulin
MDI: Maternally derived immunity
ρ: Spearman correlation coefficient
PBS: Phosphate-buffered saline
PCV-2: Porcine circovirus 2
STPr: Serum total protein obtained by optical refractometry
STPp: Serum total protein obtained by proteinogram technique
V: Vaccinated
NV: Not vaccinated
