# Supplementary material for: Can immunocrit be used as a monitoring tool for swine vaccination and infection studies?
Source: Porcine Health Manag. 2024 Aug 23;10:30. doi: 10.1186/s40813-024-00380-y (PMC11342561; doi:10.1186/s40813-024-00380-y)
Supplement: Supplementary file 1 — Additional file 1. [file 40813_2024_380_MOESM1_ESM.docx]

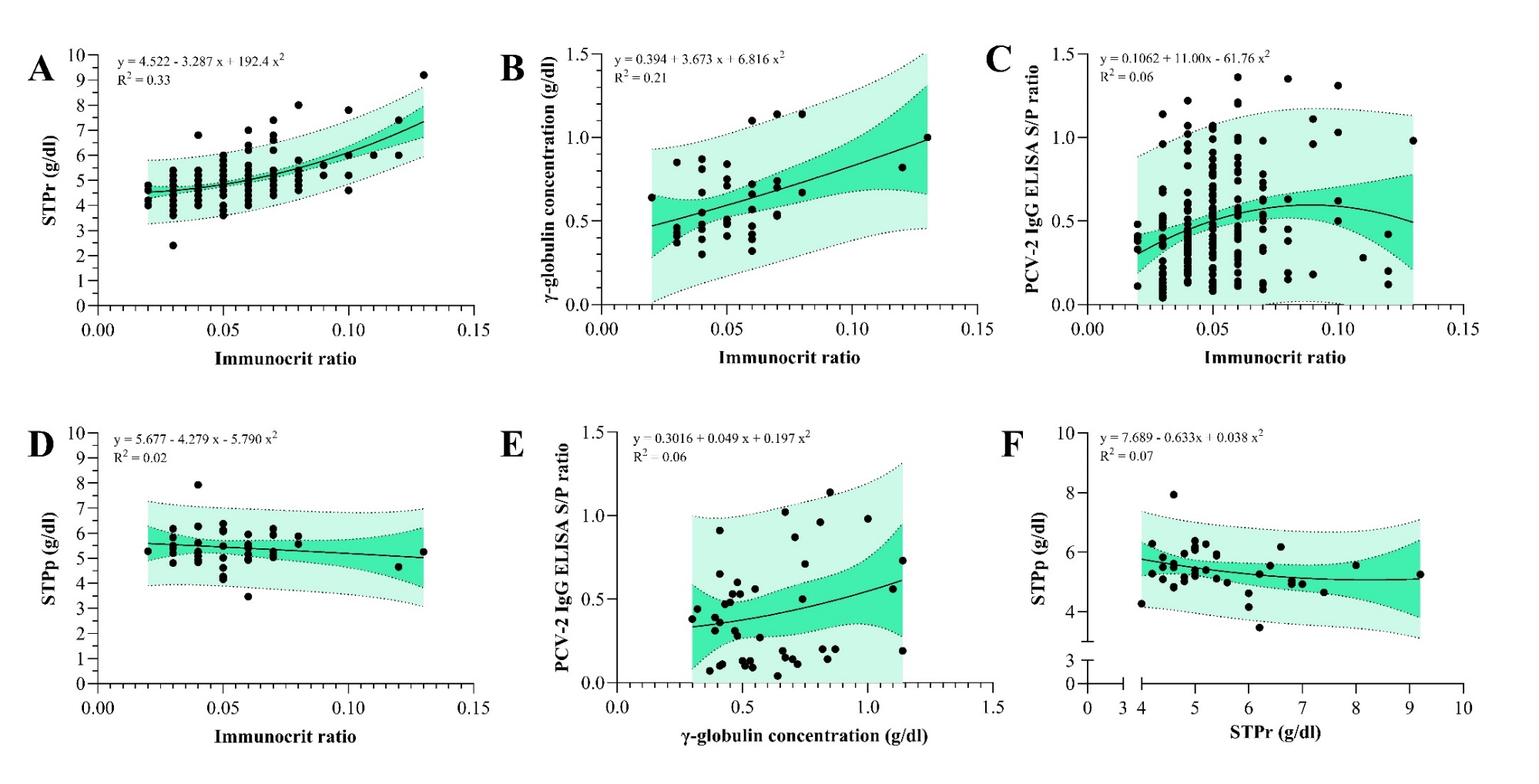


***Supplementary figure 1*.** Polynomial regression of **A)** immunocrit ratio and STPr, **B)** immunocrit ratio and γ-globulin concentration, **C)** immunocrit ratio and PCV-2 IgG ELISA S/P ratio, **D)** immunocrit ratio and STPp, **E)** γ-globulin concentration and PCV-2 IgG ELISA S/P ratio, and **F)** STPr and STPp. The upper and lower 95% confidence bands, representing the probable location of the true curve, are depicted by dashed lines filled with dark green colour. Additionally, the upper and lower 95% prediction bands, signifying the likely location of additional data points, are denoted by dashed lines filled with light green shade. STPp: serum total protein obtained by the proteinogram technique; STPr: serum total protein obtained by optical refractometry.
